# Predicting anterior cruciate ligament failure load with T_2_* relaxometry and machine learning as a prospective imaging biomarker for revision surgery

**DOI:** 10.1038/s41598-023-30637-5

**Published:** 2023-03-02

**Authors:** Sean W. Flannery, Jillian E. Beveridge, Benedikt L. Proffen, Edward G. Walsh, BEAR Trial Team, Kirsten Ecklund, Lyle J. Micheli, Brett D. Owens, Paul D. Fadale, Michael J. Hulstyn, Meggin Q. Costa, Cynthia Chrostek, Ryan M. Sanborn, Nicholas J. Sant, Yi-Meng Yen, Benedikt L. Proffen, Dennis E. Kramer, Martha M. Murray, Ata M. Kiapour, Braden C. Fleming, Dennis E. Kramer, Martha M. Murray, Ata M. Kiapour, Braden C. Fleming

**Affiliations:** 1 Department of Orthopaedics, Warren Alpert Medical School of Brown University/Rhode Island Hospital, Coro West, Suite 402, 1 Hoppin St, Providence, RI 02903 USA; 2 Division of Sports Medicine, Department of Orthopaedic Surgery, Boston Children’s Hospital, Harvard Medical School, Boston, MA USA; 3 Department of Neuroscience, Division of Biology and Medicine, Brown University, Providence, RI USA

**Keywords:** Predictive markers, Magnetic resonance imaging, Machine learning

## Abstract

Non-invasive methods to document healing anterior cruciate ligament (ACL) structural properties could potentially identify patients at risk for revision surgery. The objective was to evaluate machine learning models to predict ACL failure load from magnetic resonance images (MRI) and to determine if those predictions were related to revision surgery incidence. It was hypothesized that the optimal model would demonstrate a lower mean absolute error (MAE) than the benchmark linear regression model, and that patients with a lower estimated failure load would have higher revision incidence 2 years post-surgery. Support vector machine, random forest, AdaBoost, XGBoost, and linear regression models were trained using MRI T_2_* relaxometry and ACL tensile testing data from minipigs (n = 65). The lowest MAE model was used to estimate ACL failure load for surgical patients at 9 months post-surgery (n = 46) and dichotomized into low and high score groups via Youden’s J statistic to compare revision incidence. Significance was set at alpha = 0.05. The random forest model decreased the failure load MAE by 55% (Wilcoxon signed-rank test: p = 0.01) versus the benchmark. The low score group had a higher revision incidence (21% vs. 5%; Chi-square test: p = 0.09). ACL structural property estimates via MRI may provide a biomarker for clinical decision making.

## Introduction

Anterior cruciate ligament (ACL) injuries affect over 120,000 people in the United States each year and are especially common in adolescents^1^. Improved treatment options are needed as approximately one-third of adolescent patients will experience a graft rupture or contralateral ACL injury within 2 years post-surgery^2–6^. Furthermore, ACL reconstruction (the current gold standard) does not significantly reduce the risk of posttraumatic osteoarthritis (PTOA) relative to non-operative management^7–10^. At 20 years post-surgery, approximately 52% of ACL injured patients show radiographic signs of PTOA despite surgical treatment^8^. Successive surgeries, including revision, exacerbate PTOA risk^11^.

The causal factors for these poor outcomes are a subject of debate. Risk for revision surgery has been associated with graft choice^12^, age^13^, and incompletely restored joint kinematics^14–16^, and neuromuscular control^14–16^. A noninvasive method to evaluate the structural properties of the healing ACL or ACL graft, such as quantitative magnetic resonance imaging (qMRI), could provide insight into the mechanisms mediating reinjury and PTOA risk, guide clinical decision making, and aid in the development of improved surgical interventions (e.g., ACL restoration surgery) and rehabilitation strategies^17^.

Previously, measures obtained using qMRI (e.g., T_2_* relaxation time, signal intensity, volume, volume of organized/disorganized collagen, etc.) have been shown to relate directly to the structural properties of the healing ACL or ACL graft^18–20^, have been used to detect early soft tissue degeneration^21,22^, and have been shown to correlate with contemporaneous clinical, functional, and patient-reported outcomes^23–28^. Most recently, these qMRI measures were also shown to be associated with prospective functional outcomes following ACL restoration surgery^29^. The catastrophic clinical consequences of ACL or graft failure have driven the development of methods to predict structural properties of the healing tissue from qMRI. While linear modeling methods represent the conventional approach^18,20,30,31^, preliminary evidence suggests that machine learning techniques could provide substantial improvement to predictive performance relative to existing models as these methods have similarly shown promise in other magnetic resonance-based applications^32,33^.

The goals of this study were: (1) to train candidate machine learning models to predict post-mortem ACL failure loads based on in vivo T_2_* MR images from porcine subjects following ACL repair for which ground truth tensile failure load data were available and evaluate them relative to a benchmark linear model; and (2) to apply the best performing machine learning model to 9 month MRIs of a cohort of patients who were 2 years out from bridge-enhanced ACL restoration (BEAR) surgery and demonstrate the clinical relevance of the failure load estimates by comparing them to the incidence of revision surgery in this cohort. It was hypothesized that: (1) at least one of the machine learning models would demonstrate lower mean absolute error (MAE) on the porcine failure load data than the benchmark linear model (Aim 1); and (2) human patients with a lower estimated failure load using the machine learning model with the lowest MAE would have a higher incidence of revision surgery within 2 years post-surgery (Aim 2).

## Results

### Model development

**Figure 1:**
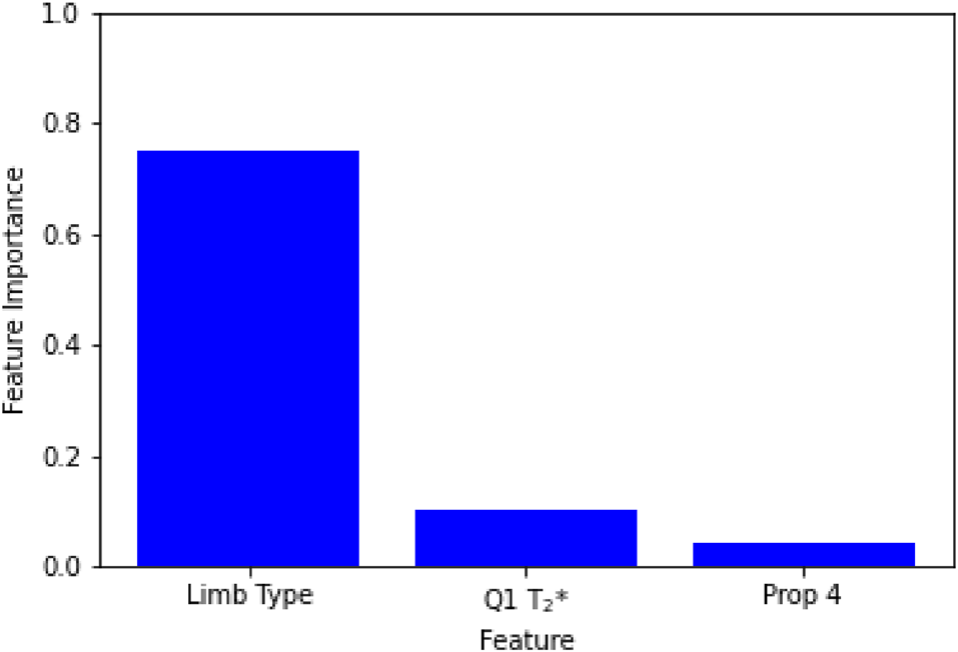
RFE-CV feature importance results (Q1 T_2_* = 1^st^ quartile T_2_*, Prop 4 = sub-volume proportion 4).

The top 3 features for the machine learning models were limb type (surgical or contralateral intact), first quartile of T_2_* relaxation time, and sub-volume proportion 4 (Fig. 1). These features were determined by RFE-CV and included in the subsequent machine learning models.

**Figure 2:**
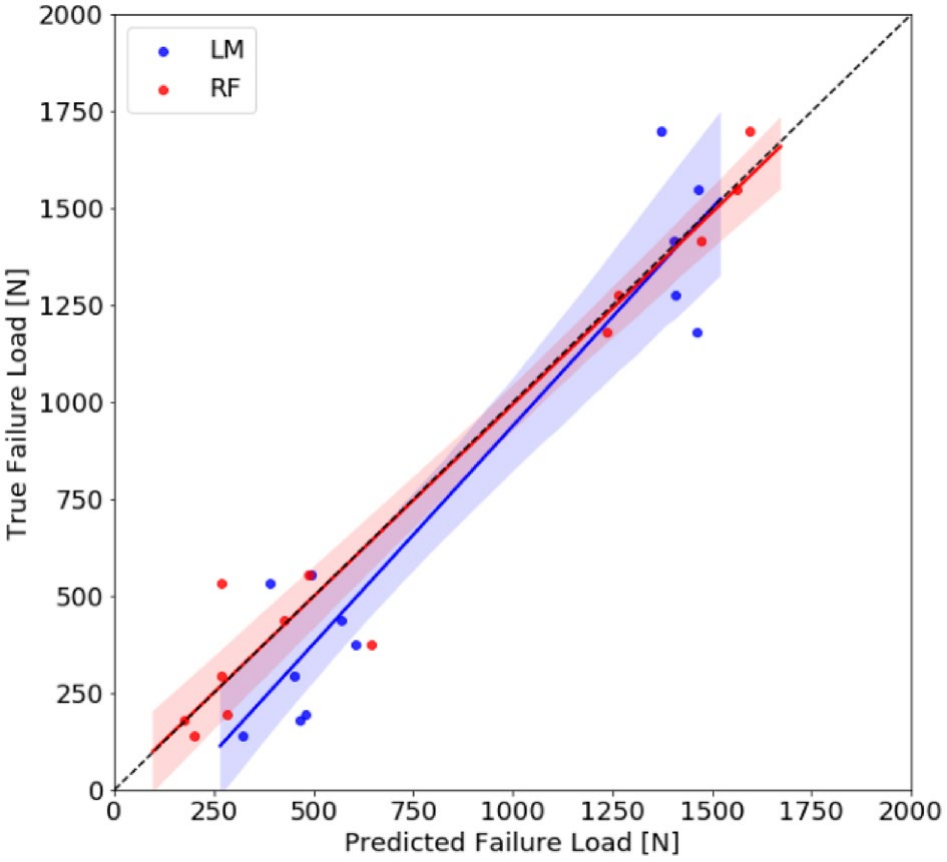
Comparison of test set predictions to ground truth failure load in Newtons (N) for best machine learning model, random forest (RF), and benchmark linear model (LM) in the porcine model. The shaded regions represent the 95% confidence intervals.

The benchmark linear regression model, which included limb type, CSA, and sub-volume proportions 1 and 4 as features, demonstrated the highest mean absolute error of all the models evaluated (Table 1). The lowest error was found using the random forest regression model. On the test set, the random forest model demonstrated a 55% (95% CI 44–78%) decrease in mean absolute error relative to the baseline linear regression model (p = 0.01; Fig. 2).

**Table 1:** Test set mean absolute error (MAE) and 95% confidence interval (CI) of the benchmark linear model (LM) and selected machine learning models (*SVM* support vector machine, *RF* random forest).

Model	MAE (95% CI) [N]
LM	178.3 (119.1, 237.4)
SVM	154.6 (110.5, 198.7)
RF	79.4 (25.9, 133.0)
AdaBoost	90.8 (40.3, 141.4)
XGBoost	136.7 (55.1, 218.3)

### Clinical pilot assessment of model performance in predicting reinjury risk

**Figure 3:**
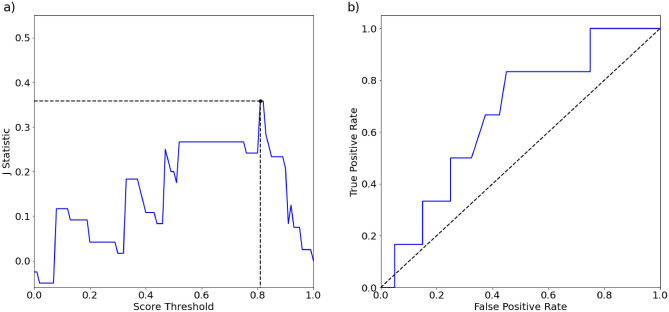
(**a**) Youden’s J statistic curve (blue) used to determine the score threshold. Optimal score threshold = 0.81 (J statistic = 0.36) is indicated by the dashed lines. (**b**) Receiver operator curve (blue) for prediction of revision surgery from load score dichotomization (area under the curve = 0.67). At the J statistic-determined threshold of 0.81, the false positive rate = 0.48 and true positive rate = 0.83.

The random forest model was then applied to the initial BEAR III patient cohort data. Based on the Youden’s J statistic, the optimal score threshold for separating the low and high failure load groups was 0.81 (Fig. 3a). At this threshold, the false positive rate was 0.48, and the true positive rate was 0.83 (Fig. 3b). For patients in the high failure load group, the incidence of revision surgery was 4.5%. For the patients in the low failure load group, the incidence was 20.8%; a 362% increase in risk for a revision surgery (likelihood ratio Chi-square test p = 0.09).

In contrast, the baseline linear model had an optimal dichotomization threshold of 0.33 as determined by Youden’s J statistic. At this threshold, the false positive rate was 0.45, and the true positive rate was 1.0 (Supplementary Table 1). For the patients in the high failure load group, the incidence of revision was 0%, and for patients in the low failure load group, the incidence was 25% (likelihood ratio Chi-square test p = 0.01).

## Discussion

All the machine learning models evaluated in this study showed decreased mean absolute errors relative to the benchmark linear regression model by 55% for predicting the tensile ACL failure load from T_2_* relaxometry data when applied to the porcine test data set (Aim 1). Of the machine learning models, the random forest regression model showed the lowest mean absolute error and was therefore used in the clinical assessment (Aim 2). When applying the random forest model to the 9-month clinical imaging data, the predicted failure loads obtained showed potential for determining which patients subsequently required revision surgery by two years as the failure rate for the high failure load group was less than 5% and that of the low failure load group was 21%.

When selecting features to include in the machine learning models, limb type (surgical or contralateral intact) was the most important feature, followed by Q1 T_2_* and sub-volume proportion 4. It was not surprising that limb type scored highly on feature importance given that feature importance tends to inflate the importance of dichotomous variables^34^. Nevertheless, limb type was considered an important variable to include in the analysis, independent of the feature importance results. Q1 T_2_* and sub-volume proportion 4 may be considered important because they relate to the most and least organized tissue within the healing ligament, respectively^30^. It has previously been shown that the quantities of the most and least organized tissue within the ACL are drivers of its structural properties^30^. Two of the features used in the baseline linear regression model were also found to be in the top 3 for feature importance (limb type and sub-volume proportion 4). Another variable used in the linear regression model, CSA, was found to be the fourth most important feature.

The best machine learning model was a random forest regression model that used limb type, Q1 T_2_*, and sub-volume proportion 4 as features. The estimated failure load predictions using the random forest approach showed a large decrease in mean absolute error (55%) in the porcine test set data compared to the benchmark linear regression that was statistically significant (p = 0.01). It is likely that this decrease is clinically meaningful, as the random forest model reduced the estimated failure load error from 31% (for the linear regression model) to 10% of the mean true failure load. Furthermore, when the model predictions were plotted versus the true failure loads, it was observed that the benchmark linear regression model overestimated low failure loads on the porcine test set, while it predicted similar values at high failure loads. Overestimating low failure loads could potentially be a limiting factor for the clinical translation of the linear model because patients with low failure loads would theoretically be the most vulnerable to reinjury. In contrast, the random forest model was more consistent across the failure load distribution.

When making estimated failure load predictions on the BEAR III patients using T_2_* relaxometry (Aim 2), a large increase in revision rate was observed for patients in the low failure load score group compared to patients in the high failure load score group. In the high failure load score group, 1 out of 22 patients required revision surgery. In the low failure load score group, 5 out of 24 patients required revision surgery. Due to the small sample size of the clinical cohort, this difference was not found to be statistically significant, though suggests a trend (p < 0.09). These findings are similar to that of the linear model, in which 0 out of 22 high failure load score patients and 6 out of 24 low failure load score patients required revision. Given that this difference was dependent on the classification of a single patient, and that the overall incidence of revision was low, more data are needed to further assess the clinical utility of the machine learning model.

Previously, we developed models predicting porcine ACL structural properties, specifically the maximum failure load, yield load, and stiffness of the ACL^18,20,30,31^. However, translating these structural property prediction models to human patients and establishing their clinical relevance was necessary. In one of our studies, we showed that estimated failure load from Constructive Interference in Steady State scans (using a different prediction model) was significantly associated with functional outcomes for patients after the ACL restoration procedure^29^. To our knowledge, the present analysis is the first to show that the T_2_* relaxometry-based structural property predictions are associated with the incidence of revision surgery and is a critical step towards translating the large body of preclinical work that we have pursued. To this end, these findings provide proof-of-concept evidence that estimating ACL failure load post-surgery using a random forest machine learning model to analyze T_2_* relaxometry images may serve as a clinically relevant biomarker to prospectively evaluate ACL surgery revision risk. This biomarker could potentially be used in the clinic to inform return-to-sport decisions and to tailor rehabilitation strategies. Future studies could be designed to evaluate the use of these failure load estimates to alter the course of healing in patients with low predicted failure loads. In research settings, noninvasively measuring the structural properties of the ACL using T_2_* relaxometry would also be a valuable tool for many research applications, for example, assessing new surgical interventions against the gold standard treatments.

There are a few study limitations to consider. First, model performance has yet to be assessed on ACL reconstructed patients. ACL reconstruction is a more common procedure, and application of this imaging approach to healing ACL grafts will be pursued in future work. As previously noted, feature importance is known to inflate the importance of dichotomous variables, such as limb type. However, for the purposes of feature selection, the magnitudes of relative feature importance values were less important than the ranking. Furthermore, limb type would be considered an important feature to include regardless of feature importance value due to differences between the healthy and repaired ligament. In addition, model specific feature importance may vary to some extent from model to model. For this reason, the main purpose of the RFE-CV procedure was to serve as a dimension reduction technique to reduce the risk of overfitting the machine learning models. For obvious ethical reasons, in vivo ground truth ACL failure load data were not available for human patients. As a result, a direct validation of the model failure load predictions could not be performed on patients, and thus were derived from porcine data, which were then normalized and scaled for human use. Therefore, the clinical impact of the estimated failure load scores was assessed by relating model predictions of failure load at 9 months (the time at which patients were permitted to return-to-sport^35,36^) to revision surgery rates by 2 years. Furthermore, by converting the failure load prediction to a 0–1 score, the metric was normalized to the range of possible estimated failure load values, reducing the importance of the actual absolute value. This approach also has the benefit of providing a more easily interpretable metric for clinical use. Another limitation was that the porcine data used for the initial model training were acquired with a 6-channel coil, rather than the 15-channel coil used for the human data. Because the 6-channel coil has a lower signal-to-noise ratio than the 15-channel coil, its use would potentially make the machine learning prediction model more robust by acting as a form of noise augmentation^37,38^. Finally, the overall incidence of revision in the BEAR III cohort study was low (6 out of 46), which may explain the relatively high false positive rate. The incidence of revision surgery was chosen as a benchmark for comparison in the human study because it is a discrete outcome and clinically highly relevant. However, there are other clinically relevant outcomes that the model should be compared to, such as incidence of PTOA. This could be assessed as long-term follow up data become available from the clinical trial analyzed in the present study. The sample sizes for the animal and clinical studies were based on samples of convenience as the current analysis leveraged existing data sets. Nonetheless, the sample size was large enough to show that the predicted failure load score from T_2_* images may be related to risk of reinjury and provides proof-of-concept evidence that it could serve as a biomarker to predict ACL reinjury. Future studies involving a larger independent test cohort are needed to fully validate the method.

In conclusion, the best machine learning model (random forest regression) predicted the ACL failure load in porcine subjects with lower error and lower bias than the benchmark linear regression model. The application of the random forest regression model to human T_2_* data at 9 months post-surgery shows a potential relationship with the incidence of revision surgery out to 2 years, where patients with a higher estimated failure load score showed a decreased incidence of revision surgery, though more data are needed to support these clinical pilot results. Estimating structural properties of the ACL post-surgery may therefore be a relevant biomarker for both clinical and research applications.

## Methods

### Model development

#### Porcine data

**Figure 4:**
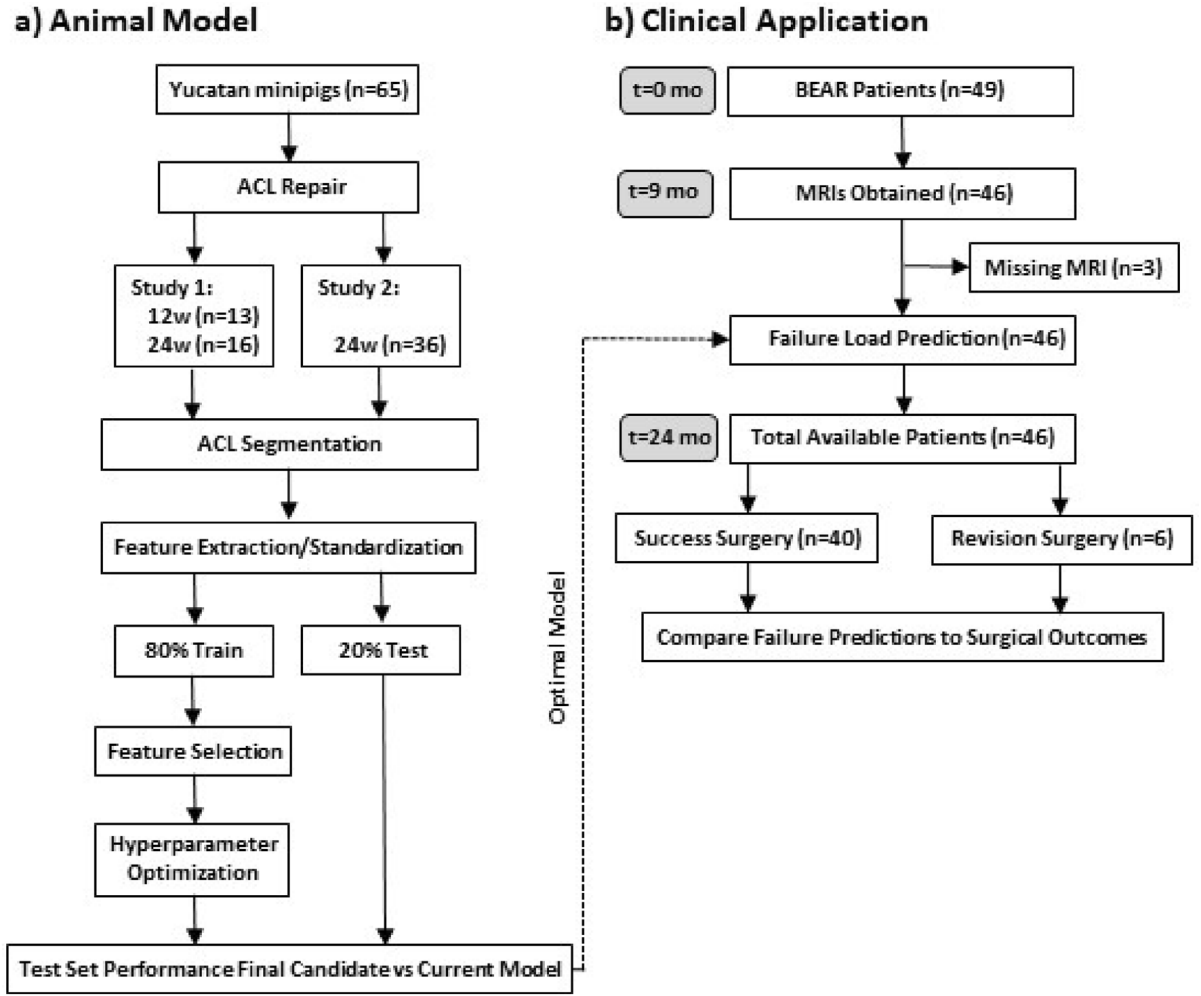
(**a**) Animal study to evaluate the five prediction models to select the one with the lowest mean absolute error (MAE). (**b**) STROBE diagram detailing the available BEAR III trial data analysis using the best prediction model determined in the animal study (dashed arrow). *ACL* anterior cruciate ligament, *BEAR* Bridge-enhanced ACL Restoration, *t* time, *mo* month.

Data were acquired from two previous studies of ACL transection followed by surgical repair with the BEAR implant in Yucatan minipigs (Fig. 4A)^30,31^. These studies were performed to meet the ARRIVE guidelines and were IACUC approved. In the first study, paired MRI and tensile failure load data of the ACL repaired limb and contralateral uninjured limb were available at 12 (n = 13) and 24 weeks post-surgery (n = 16)^30,31^. In the second study, the same data were available at 24 weeks post-surgery (n = 36)^31,39^. In total, paired MRI and tensile failure load data were available for 65 minipigs.

For the pig studies, MRI T_2_* relaxometry scans were acquired using a 3 T magnet (PRISMA, Siemens, Erlangen, Germany) with a 6-channel flexible coil (Flexcoil; Siemens), and a gradient echo 4-echo sequence (FA = 12°; TR = 29 ms; TE_1_ = 2.8 ms, TE_2_ = 7.9 ms, TE_3_ = 13 ms, TE_4_ = 18 ms; FOV = 160 mm; 512 × 512 acquisition matrix, voxel size of 0.3125 mm × 0.3125 mm × 0.8 mm)^30,40^. Both the surgical and contralateral knees were imaged. Following in vivo imaging of both knees, the animals were euthanized, and the hind limbs were harvested and frozen at − 20 °C until mechanical testing^30,31^. The porcine data were randomly split and stratified by subject into an 80% training (n = 52) set and a 20% test (n = 13) set. Given the small sample size, a cross validation within the training set was performed as it is more robust than an independent validation set^38^.

#### MR image processing

The ACL was manually segmented from the image volume by one segmenter with 4 years of experience (SWF) using commercial imaging software (Mimics Research 19.0; Materialise, Leuven, Belgium). Voxel-wise T_2_* relaxation times were calculated by fitting an exponential decay curve to the per echo signal intensities^41^. Based on our previous studies, the following MR variables were extracted for potential inclusion in the prediction models: median T_2_*, mean T_2_*, standard deviation T_2_*, skew T_2_*, first quartile (Q1) T_2_*, third quartile (Q3) T_2_*, average cross-sectional area (CSA), and sub-volume proportions (Prop 1–4)^18,30^. Average CSA was estimated as ligament volume divided by ligament length. Four sub-volume proportions were identified by binning voxels by relaxation time (0–12.5 ms, 12.5–25 ms, 25 ms, 25–37.5 ms, 37.5–50 ms) and dividing by the total ligament volume. All these variables were selected because they are either ligament size-invariant or ligament size-normalized. It was expected that these variables would scale more readily from pigs to humans. All variables were standardized by z-scoring prior to use by the machine learning models.

#### Biomechanical testing

**Figure 5:**
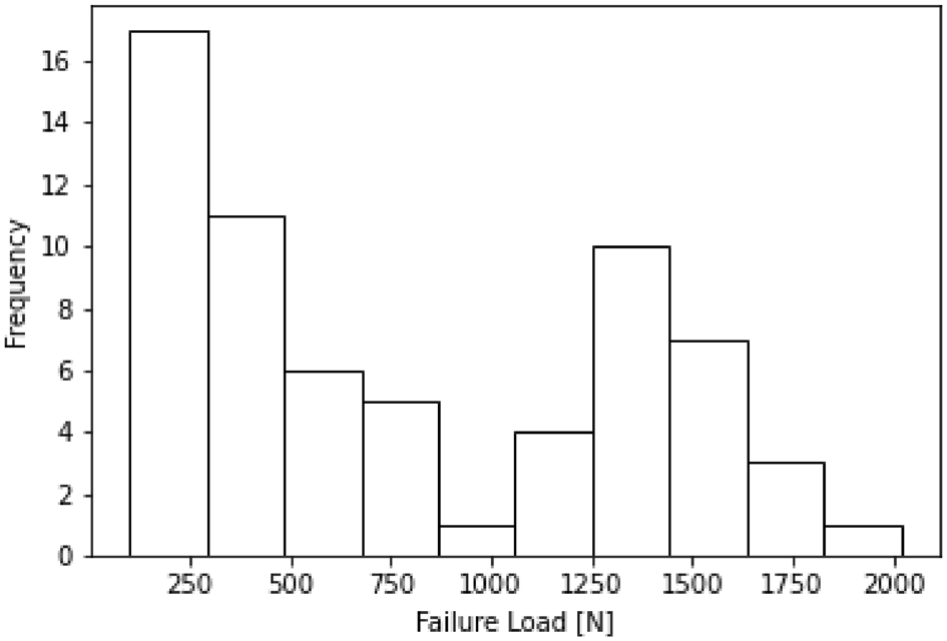
Histogram showing the range of failure loads measured during the biomechanical testing of the porcine limbs.

The porcine hind limbs were thawed to room temperature prior to biomechanical testing. The joints were dissected down to the femur-ACL-tibia complex, then the femur and tibia were potted in PVC pipes and secured with a urethane resin (SmoothCast; Smooth-On, Macungie, PA). Each sample was mounted for mechanical testing in a servo-hydraulic material testing system (MTS 810, Eden Prairie, MN) such that the long axis of the ACL was aligned with the tensile load vector. Tensile loading was then applied at a rate of 20 mm/min until failure^31^. Failure load, yield load, and stiffness were calculated for each specimen as previously described^30^. Given the high correlation between these three variables^18,20,30^, only failure load was evaluated in the present study (Fig. 5).

#### Machine learning model development

Multiple machine learning regression models were developed and compared to predict the ACL failure load: support vector machine (SVM), random forest (RF), AdaBoost, and XGBoost (Table 2)^38^. A multivariate linear regression model (LM) was trained as the performance benchmark. Model selection was influenced, in part, by the sample size available for training and evaluation^38^. The SVM regressor was trained by fitting a hyperplane to the data that maximized instances between the margins and minimized violations outside of the margins. Random forest, AdaBoost, and XGBoost were variations of decision tree ensembles. The random forest model was a parallel ensemble of decision trees trained on random samples of data selected with replacement (i.e., bagging)^38^. AdaBoost and XGBoost both combined multiple weak models sequentially to create a single strong learner (i.e., boosting)^38^. During this process, more weight was placed on difficult instances so that subsequent learners improved predictions on those instances. XGBoost also added regularization (L1, L2, pruning) to reduce the risk of overfitting^38^.

**Table 2:** Initial hyperparameter search space (*SVM* support vector machine, *RF* random forest, *RBF* radial basis function, *MSE* mean squared error, *DART* dropouts meet multiple additive regression trees).

Model	Hyperparameter	Search range
SVM	C	0.1:1000
	Kernel	RBF, polynomial, sigmoid, linear
	Degree	1:6
	Gamma	0.0001:1
RF	N estimators	100:2000
	Criterion	MSE, MAE
	Max depth	5:100
	Max features	Auto, Square Root, Log_2_
	Min samples leaf	1:5
	Min samples split	2:10
	Bootstrap	True, false
AdaBoost	N estimators	100:2000
	Learning rate	0.1:2
	Loss	Linear, square, exponential
XGBoost	N estimators	100:2000
	Max depth	5:100
	Booster	Gblinear, DART
	Gamma	0:200
	Learning rate	0.01:0.7
	Regularization alpha	0:200
	Regularization lambda	0:200

#### Feature selection

In addition to the MR imaging variables extracted, limb type (surgical or contralateral intact) was also included as a potential variable. The benchmark linear model used sub-volume proportions 1 and 4, CSA, and limb type as features. This linear model was an evolution of a previously published linear model relating T_2_* relaxation times to the structural properties^30^ but was based on size-normalized features in the current study. Features for the machine learning models were selected via recursive feature elimination with cross-validation (RFE-CV)^42,43^. In the RFE-CV procedure, an estimator was initially fitted on the training set using all possible features and fivefold cross-validation. Cross-validation ensured that the feature importance estimation was robust to the data split used, by iteratively withholding a different subset of the data for validation^38^. After cross-validation, the least important feature was eliminated, and the process was repeated with the minimum number of features at the finish set to 3. This threshold was selected because of the rapid decay in feature importance levels beyond 3 features.

#### Model optimization and selection

Each machine learning model was optimized by first performing a random search fivefold cross-validation (RS-CV) to narrow the possible hyperparameter space (Table 2). Next, a grid search cross-validation (GS-CV) was performed in the reduced hyperparameter space. This approach was more computationally efficient than performing GS-CV on the full hyperparameter space^38^. After model optimization, final performance of each machine learning model was assessed by making predictions on the withheld test set. The best (lowest error) machine learning model was compared to the benchmark linear model using the mean absolute error values from these test set predictions. The statistical significance of the difference in mean absolute error was assessed with a Wilcoxon signed-rank test.

### Clinical pilot assessment of model performance in predicting reinjury risk

#### Data set

MRI T_2_* data were acquired from patients enrolled in the ongoing, IRB-approved BEAR III clinical trial (NCT03348995) and who were at least 2 years post-surgery (Fig. 4B)^44^. The BEAR III trial was approved by the Institutional Review Boards of Boston Children’s Hospital and Rhode Island Hospital, and all subjects granted their informed consent. Eligible patients received ACL restoration surgery within 50 days of ACL injury using the BEAR implant (Boston Children’s Hospital, Boston MA), a scaffold composed of bovine-derived extracellular matrix proteins^45,46^. The inclusion criteria were male and female patients, 12–80 years of age, a first-time complete mid-substance ACL tear or partial tear accompanied by a positive pivot shift. Patients were included if they had at least 5% of ACL length remaining on the tibia, as determined via MRI. Subjects who tore their contralateral ACL, re-tore their ipsilateral ACL, or had missing data at any timepoint were excluded from the present analysis. Two-year data were available for 49 subjects (Fig. 4B; 25 female, 24 male; mean age 21 [range 13–47] years)^44^. Three patients missed their 9-month MRI exams, and no patients were lost to follow up, making 46 subjects available for the present analysis. During the 2-year post-surgery period, a total of 6 ACL injuries requiring revision surgery occurred.

MR imaging was performed 9 months post-surgery using either a 3 T Prisma (Siemens, Erlangen, Germany) or a 3 T Tim Trio (Siemens) scanner with a 15-channel transmit/receive coil (Siemens). On the Prisma (Brown University; n = 10), T_2_* relaxometry scans were acquired with FA = 12°; TR = 29 ms; TE_1_ = 2.5 ms; TE_2_ = 6.9 ms; TE_3_ = 11.2 ms; TE_4_ = 15.6 ms; TE_5_ = 20 ms; TE_6_ = 24.4 ms; FOV = 160 mm; 384 × 384 acquisition matrix with voxel size 0.4167 mm × 0.4167 mm × 0.8 mm, acquisition time 11 min 57 s. On the Tim Trio (Boston Children’s Hospital Waltham, n = 36), T_2_* relaxometry scans were acquired with FA = 12°; TR = 29 ms; TE_1_ = 3.4 ms; TE_2_ = 6.9 ms; TE_3_ = 11.2 ms; TE_4_ = 15.6 ms; TE_5_ = 20 ms; TE_6_ = 24.4 ms; FOV = 160 mm; 384 × 384 acquisition matrix with voxel size 0.4167 mm × 0.4167 mm × 0.8 mm, acquisition time 11 min 59 s. Due to the acquisition parameter differences between scanners imposed by the differing gradient hardware performance limits, all acquisitions were harmonized prior to analysis by z-scoring^47^. Details regarding the MRI harmonization procedure have been previously described^47^.

#### Clinical application of the optimal machine learning model

The machine learning model with the lowest MAE as determined in Aim 1 was used to estimate the failure load of the healing ACL at 9 months post-surgery for the patients treated with the BEAR implant. Prior to making predictions, each continuous feature in the MR data that was included in the model was standardized by z-scoring with the mean and standard deviation of the same feature from pigs used as the scaling reference. This standardization step was necessary to ensure the patient imaging data were on the same scale as the porcine data that the model was trained on.

Failure load predictions were converted to a score from 0–1 (Eq. 1; F_failure_ = estimated failure load of subject, F_min_ = lowest estimated failure load in dataset, F_max_ = highest estimated failure load dataset). A score threshold was objectively determined using the Youden’s J statistic (Eq. 2; TP = true positive, TN = true negative, FN = false negative, FP = false positive)^48^. Patients with a predicted failure load score less than or equal to a score threshold were assigned to the low failure load group (i.e., at high risk for reinjury), and those that were greater than the score threshold assigned to the high failure load group (i.e., at lower risk for reinjury). For a dichotomous variable, the optimal threshold was located where the J statistic was maximized^48^. The difference in revision rates within 2 years of surgery between the MRI predicted low and high failure load groups was then assessed with a likelihood ratio Chi-square test. The level of significance was set at alpha = 0.05 for all statistical tests.

1 $$Estimated \; Failure \; Load \; Score= \frac{{F}_{failure}-{F}_{min}}{{F}_{max}-{F}_{min}},$$

2 $$J= \frac{TP}{TP+FN}+\frac{TN}{TN+FP}-1.$$

## Supplementary Information

Supplementary Table 1.

## Data Availability

Please contact the corresponding author for data access.
