# Supplementary material for: Predicting anterior cruciate ligament failure load with T2* relaxometry and machine learning as a prospective imaging biomarker for revision surgery
Source: Sci Rep. 2023 Mar 2;13:3524. doi: 10.1038/s41598-023-30637-5 (PMC9981601; doi:10.1038/s41598-023-30637-5)
Supplement: Supplementary file 1 — Supplementary Table 1. [file 41598_2023_30637_MOESM1_ESM.pdf]

Predicting Anterior Cruciate Ligament Failure Load with T_2* Relaxometry and Machine Learning as a Prospective Imaging Biomarker for Revision Surgery

Sean W. Flannery, Jillian E. Beveridge, Benedikt L. Proffen, Edward G. Walsh, BEAR Trial Team, Dennis E. Kramer, Martha M. Murray, Ata M. Kiapour, Braden C. Fleming

Supplementary Table 1. Clinical pilot study results of all models (RF=random forest, LM=linear model, SVM=support vector machine, TPR=true positive rate, FPR=false positive rate).

Model	Score Threshold	J Statistic	TPR	FPR	High Score Revision	High Score Survive	Low Score Revision	Low Score Survive
RF	0.81	0.36	0.83	0.48	1	21	5	19
LM	0.33	0.55	1.00	0.45	0	22	6	18
AdaBoost	0.95	0.23	0.33	0.10	4	36	2	4
XGBoost	0.39	0.21	0.33	0.13	4	35	2	5
SVM	0.81	0.20	0.50	0.30	3	28	3	12
